# Outcomes and mortality predictors after tracheostomy in intensive care unit patients with infectious diseases: A 5-year retrospective cohort study from Brazil

**DOI:** 10.1038/s41598-026-51458-2

**Published:** 2026-05-08

**Authors:** André Figueiredo Accetta, Denise Machado Medeiros, Alex Mendonça Chaves, Maria Pia Diniz Ribeiro, Sandra Wagner Cardoso, Isabel Cristina Ferreira Tavares, Valdilea Gonçalves Veloso, André Miguel Japiassú, Hugo Boechat Andrade

**Affiliations:** 1 School of Medicine, Universidade Federal Fluminense (UFF), Niterói, RJ Brazil; 2 Instituto Nacional de Infectologia Evandro Chagas (INI), Fundação Oswaldo Cruz (Fiocruz), Rio de Janeiro, RJ Brazil; 3 Instituto Biomédico, Universidade Federal Fluminense (UFF), Niterói, RJ Brazil

**Keywords:** Tracheostomy, Infectious diseases, Mortality, Prognosis, Intensive care units, Diseases, Health care, Medical research, Risk factors

## Abstract

Tracheostomy is frequently performed in critically ill patients requiring prolonged mechanical ventilation, but evidence regarding its prognostic impact in infectious diseases remains limited. This retrospective cohort study analyzed 607 patients who underwent tracheostomy at a specialized infectious disease hospital in Brazil between May 2020 and April 2025. Demographic data, comorbidities, severity scores, perioperative variables, and clinical outcomes were extracted from medical records, and multivariate logistic regression identified factors independently associated with in-hospital mortality. Overall hospital mortality was 64.6%. Age over 60 years, diabetes mellitus, higher SAPS 3 score, coagulopathy, need for hemodialysis, and a PaO₂/FiO₂ ratio below 200 on the day of tracheostomy were independently associated with death. In a sensitivity analysis excluding patients with COVID-19, HIV infection (analyzed as an underlying comorbidity) was associated with increased mortality, while the use of combined antiretroviral therapy was associated with improved survival; these results should be interpreted as exploratory. Overall, outcomes after tracheostomy in patients with infectious diseases appear to be driven primarily by systemic severity and organ dysfunction rather than by infectious etiology, underscoring the importance of individualized, multiparametric prognostic assessment.

**Supplementary Information:**

The online version contains supplementary material available at 10.1038/s41598-026-51458-2.

## Introduction

Infectious diseases remain one of the leading causes of morbidity and mortality worldwide, particularly when they progress to acute respiratory compromise. Infectious agents such as respiratory viruses, bacteria, and fungi can trigger severe pneumonia, acute respiratory distress syndrome (ARDS) and pulmonary sepsis^1,2^. In these conditions, patients may develop hypoxemic or hypercapnic respiratory failure, requiring invasive ventilatory support in the intensive care unit^3,4^.

However, in patients requiring prolonged mechanical ventilation, maintaining an artificial airway through orotracheal intubation is associated with significant complications, including laryngeal injury, patient discomfort, need for continuous sedation, and increased risk of ventilator-associated pneumonia^5,6^. In this context, tracheostomy emerges as an effective alternative to improve airway management^7,8^.

In recent years, particularly during the COVID-19 pandemic, there has been a marked increase in the number of tracheostomies performed in patients with severe infectious diseases. Studies conducted in this setting have demonstrated that tracheostomy can be performed safely when appropriate technical standards and protection protocols are followed^9,10^. However, these findings primarily address procedural safety regarding biological risk to healthcare workers and should not be directly extrapolated to patient-centered clinical outcomes.

Previous studies, including data from COVID-19 cohorts^11,12^, have suggested that clinical severity plays a central role in determining outcomes after tracheostomy. The present study expands this analysis to a broader population of critically ill patients with infectious diseases.

Patients with infectious diseases represent a complex ICU population, in which outcomes are influenced not only by the severity of respiratory failure but also by pathogen-related lung injury, systemic inflammation, immunosuppression, and infection control considerations^2,3^. These factors may directly impact both the indication and timing of tracheostomy, as well as patient prognosis.

Despite its widespread use, controversies persist regarding tracheostomy in critically ill patients. Potential benefits include reduced duration of mechanical ventilation and lower rates of ventilator-associated pneumonia^13^, although consistent evidence of mortality reduction is lacking^14^. In addition, the procedure carries risks such as complications, increased morbidity, and uncertainties related to optimal timing and patient selection^9^.

Therefore, this study aimed to evaluate clinical outcomes and identify predictors of in-hospital mortality among intensive care unit patients undergoing tracheostomy during the management of infectious diseases in a referral hospital. By analyzing a large cohort over a five-year period, this study seeks to contribute to a better understanding of prognostic factors associated with outcomes in this complex population.

## Results

Of a total of 8,633 hospitalized patients, 4,624 (53.5%) were admitted to the intensive care unit. Among them, 607 patients underwent tracheostomy in the context of infectious diseases, and none were excluded from the study. Of the operated patients, 215 (35.4%) survived and were followed until hospital discharge, while 392 (64.6%) died. Four patients required emergency tracheostomy due to failed intubation.

The demographic and clinical characteristics of the cohort are presented in Table 1. The median age was 55 years, and 64% of the patients were male. Patients who died were significantly older than survivors (median age 60 vs. 47 years, *p* < 0.01). Clinical severity scores at admission were also higher among nonsurvivors: SAPS 57 (IQR 49–71) vs. 52 (IQR 44–66), *p* < 0.01; and SOFA 5 (IQR 3–8) vs. 4 (IQR 2–7), *p* < 0.01.

**Table 1:** Demographic and clinical characteristics of 607 ICU patients undergoing tracheostomy for infectious diseases in a referral center in Brazil (2020–2025).

Proportion or median	All	Survivors	Non-survivors		*p*-value
	*N* = 607 (100%)	*N* = 215 (35.4%)	*N* = 392 (64.6%)		
*Demographic characteristics*					
Age years (median, interquartile range)	55 (41–67)	47 (37–61)	60 (45–70)	*p* < 0.01^2^	
Sex at birth (male)	389 (64)	146 (67.9)	243 (61.9)	*p* = 0.15^1^	
Body mass index (median, interquartile range)	25 (20–30)	25 (19–30)	25 (20–29)	*p* = 0.68^2^	
Days of symptoms^a^, days (median, interquartile range)	7 (3–10)	7 (4–11)	7 (3–10)	*p* = 0.22^2^	
*Comorbidities*					
Hypertension	258 (42.5)	77 (35.8)	181 (46.1)	*p* = 0.01^1^	
HIV infection^b^	185 (30.4)	71 (33.0)	114 (29.0)	*p* = 0.31^1^	
Diabetes	111 (18.2)	23 (10.6)	88 (22.4)	*p* < 0.01^1^	
Chronic pneumopathy	72 (11.8)	19 (8.8)	53 (13.5)	*p* = 0.09^1^	
Congestive heart disease	17 (2.8)	5 (2.3)	12 (3.0)	*p* = 0.60^1^	
Neoplastic disease	9 (1.4)	2 (0.9)	7 (1.7)	*p* = 0.40^1^	
Hemodialysis (previous)	8 (1.3)	2 (0.9)	6 (1.5)	*p* = 0.54^1^	
*Severity scores*					
SAPS 3, points (median, interquartile range)	55 (47–70)	52 (44–66)	57 (49–71)	*p* < 0.01^2^	
SOFA, points (median, interquartile range)	4 (2–8)	4 (2–7)	5 (3–8)	*p* < 0.01^2^	

^1^Pearson. ^2^Wilcoxon. Congestive heart disease: heart functional classification by New York Heart Association - NYHA ≥ II. Chronic pneumopathy: GOLD III/IV chronic obstructive pulmonary disease. Hemodialysis (previous): end-stage renal disease on hemodialysis before admission; SAPS 3: Simplified Acute Physiology Score 3; SOFA: sequential organ failure assessment score.

^a^Onset to hospital admission.

^b^HIV infection was considered an underlying chronic condition.

Regarding comorbidities, diabetes and hypertension were significantly more frequent among patients who died (22.4% and 46.1%, respectively; *p* < 0.01). Other variables, including body mass index, time from symptom onset to hospital admission, presence of chronic lung disease, congestive heart failure, malignancy, prior hemodialysis, and sex at birth, showed no statistically significant differences between groups.

The infectious diseases identified in the cohort are summarized in Table 2. A wide range of conditions was observed, with COVID-19 being the most frequent (364 cases; 59.9%), followed by tuberculosis (23.2%). HIV infection, analyzed as an underlying comorbidity, was present in 30.4% of the total population.

**Table 2:** Distribution of infectious diseases and infection-related conditions among 607 ICU patients undergoing tracheostomy for infectious diseases in a referral center in Brazil (2020–2025).

Proportion or median	ALL	Survivors	Non-survivors	*p*-value
	*N* = 607 (100%)	*N* = 215 (35.4%)	*N* = 392 (64.6%)	
COVID-19	364 (59.9)	118 (54.8)	246 (62.7)	*p* = 0.06^1^
Tuberculosis^a^	141 (23.2)	62 (28.8)	79 (20.2)	*p* < 0.01^1^
Pneumocystis carinii	52 (8.5)	23 (10.6)	29 (7.3)	*p* = 0.16^1^
Toxoplasmosis	36 (5.9)	14 (6.5)	22 (5.6)	*p* = 0.65^1^
Meningitis	22 (3.6)	10 (4.6)	12 (3.0)	*p* = 0.32^1^
Syphilis	21 (3.4)	10 (4.6)	11 (2.8)	*p* = 0.23^1^
Histoplasmosis	12 (1.9)	5 (2.3)	7 (1.7)	*p* = 0.65^1^
Herpes	11 (1.8)	3 (1.3)	8 (2.0)	*p* = 0.57^1^
Aspergillosis	9 (1.4)	2 (0.9)	7 (1.7)	*p* = 0.40^1^
Cryptococcosis	9 (1.4)	3 (1.3)	6 (1.5)	*p* = 0.90^1^
Hepatitis B	6 (0.9)	4 (1.8)	2 (0.5)	*p* = 0.11^1^
Hepatitis C	5 (0.8)	1 (0.4)	4 (1.0)	*p* = 0.47^1^
Endocarditis	4 (0.6)	3 (1.3)	1 (0.2)	*p* = 0.10^1^
Mpox	2 (0.3)	1 (0.4)	1 (0.2)	*p* = 0.67^1^
Leptospirosis	2 (0.3)	1 (0.4)	1 (0.2)	*p* = 0.67^1^
Tetanus	2 (0.3)	1 (0.4)	1 (0.2)	*p* = 0.67^1^
Paracoccidioidomycosis	1 (0.1)	0 (0.0)	1 (0.2)	*p* = 0.46^1^

^1^Pearson. COVID-19: Coronavirus disease 2019 by SARS-CoV-2. HIV/AIDS: human immunodeficiency virus / acquired immunodeficiency syndrome.

^a^Tuberculosis cases included patients with severe or disseminated forms requiring intensive care.

When associations with hospital mortality were analyzed, only tuberculosis showed a statistically significant difference between groups. The prevalence of tuberculosis-related clinical illness was higher among survivors (28.8%) than among nonsurvivors (14%, *p* < 0.01). Among the comorbidities evaluated, HIV infection was not significantly associated with mortality in the primary analysis (*p* = 0.31). COVID-19 was present in 62.7% of deaths compared with 54.8% of survivors, showing a trend toward statistical significance (*p* = 0.06), although not reaching the conventional threshold. Other opportunistic and systemic infections showed no significant association with mortality; however, the low absolute frequency of some pathogens may have limited the statistical power of this analysis.

Disease severity at the time of tracheostomy and surgical complications are summarized in Table 3. Clinical assessment on the day of surgery demonstrated that coagulopathy, the need for hemodialysis, and a low PaO₂/FiO₂ (P/F) ratio were significantly associated with in-hospital mortality.

**Table 3:** Clinical status at the time of tracheostomy and surgical complications in 607 ICU patients undergoing tracheostomy for infectious diseases in a referral center in Brazil (2020–2025).

Proportion or median	All	Survivors	Non-survivors	*p*-value
	*N* = 607 (100%)	*N* = 215 (35.4%)	*N* = 392 (64.6%)	
Vasopressor use on surgery day	350 (57.6)	116 (53.9)	234 (59.6)	*p* = 0.17^1^
Coagulation issue on surgery day^a^	79 (13.0)	14 (6.5)	65 (16.5)	*p* < 0.01^1^
Hemodialysis on surgery day	210 (34.5)	47 (21.8)	163 (41.5)	*p* < 0.01^1^
P/F on surgery day (median, interquartile range)	301 (210–447)	353 (237.5-496.7)	277.5 (192.2-416.6)	*p* < 0.01^2^
Postoperative complications (yes)^b^	40 (6.5)	18 (8.3)	22 (5.6)	*p* = 0.01^1^
• Hemorrhage		4	13	
• Accidental decannulation		2	7	
• Ventilatory problem		2	6	
• Others		6	4	
• Stenosis		4	0	

^1^Pearson. ^2^Wilcoxon. MV: mechanical ventilation; P/F: the ratio of arterial oxygen partial pressure to fractional inspired oxygen; TCT: tracheostomy.

^a^Coagulation issue was defined as: thrombocytopenia (< 100,000), or prolonged Prothrombin Time (PT) and/or activated Partial Thromboplastin Time (aPTT); or active bleeding and/or the need for plasma and/or platelet transfusions for the surgical procedure.

^b^Patients may present more than one postoperative complication.

Coagulopathy was present in 16.5% of patients who died compared with 6.5% of survivors (*p* < 0.01), while hemodialysis on the day of tracheostomy was observed in 41.5% of nonsurvivors versus 21.8% of survivors (*p* < 0.01). The median P/F ratio was lower among patients who died (277.5; IQR 192.2–416.6) than among survivors (353; IQR 237.5–496.7; *p* < 0.01). Other variables, including the use of vasoactive agents, showed no statistically significant association with mortality.

Clinical outcomes are summarized in Table 4. The median length of hospital stay for the cohort was 42 days (IQR 29–66). Survivors had higher decannulation rates (90.6% vs. 2.8%, *p* < 0.01), longer duration of tracheostomy (median 28 vs. 14 days, *p* < 0.01), and longer hospital (62 vs. 34 days, *p* < 0.01) and ICU stays (40 vs. 31 days, *p* < 0.01). These findings reflect differences related to survival and should be interpreted as descriptive outcomes rather than causal associations.

**Table 4:** Clinical outcomes of 607 ICU patients undergoing tracheostomy for infectious diseases in a referral center in Brazil (2020–2025).

Proportion or median	All	Survivors	Non-survivors	*p*-value
	*N* = 607 (100%)	*N* = 215 (35.4%)	*N* = 392 (64.6%)	
Decannulated patients	206 (33.9)	195 (90.6)	11 (2.8)	*p* < 0.01^1^
Duration of TCT^a^, days (median, interquartile range)	19 (10.0-35.5)	28 (18.0-41.8)	14 (7.0-29.2)	*p* < 0.01^2^
Duration of MV, days (median, interquartile range)	29 (23–41)	28 (22–39)	30 (23–43)	*p* = 0.06^2^
LOS^a^ INI ICU, days (median, interquartile range)	35 (26–49)	40 (30.2–52.0)	31 (24–45)	*p* < 0.01^2^
LOS^a^ INI, days (median, interquartile range)	42 (29–66)	62.0 (46.2–87.8)	34 (25–51)	*p* < 0.01^2^
Mortality ICU	361 (59.4)	0 (0.0)	361 (92.0)	*p* < 0.01^1^

^1^Pearson. ^2^Wilcoxon. ICU: intensive care unit; INI: Instituto Nacional de Infectologia; LOS: length of stay; MV: mechanical ventilation; TCT: tracheostomy.

^a^Comparisons for length of stay (LOS) and tracheostomy (TCT) duration are provided for descriptive purposes and should be interpreted considering the competing risk of mortality.

The results of the logistic regression analysis for in-hospital mortality are presented in Table 5. Multivariate logistic regression identified independent predictors of in-hospital mortality, including age over 60 years (OR 2.19; 95% CI 1.38–3.49; *p* = 0.001), diabetes mellitus (OR 2.07; 95% CI 1.20–3.67; *p* = 0.010), higher SAPS score (OR 1.92; 95% CI 1.20–3.10; *p* = 0.007), presence of coagulopathy on the day of surgery (OR 2.60; 95% CI 1.39–5.15; *p* = 0.004), hemodialysis on the day of surgery (OR 2.47; 95% CI 1.61–3.82; *p* < 0.001), and PaO₂/FiO₂ ratio < 200 (OR 2.62; 95% CI 1.62–4.35; *p* < 0.001).

**Table 5:** Multivariate logistic regression analysis of independent predictors associated with in-hospital mortality in 607 ICU patients undergoing tracheostomy for infectious diseases in a referral center in Brazil (2020–2025).

Factor	OR	95% CI	*p*-value
Age > 60 years	**2.19**	**1.38 to 3.49**	**0.001**
COVID-19	1.18	0.68 to 2.04	0.555
Diabetes	**2.07**	**1.20 to 3.67**	**0.010**
Hypertension	0.98	0.63 to 1.55	0.945
HIV infection	1.26	0.74 to 2.14	0.391
SAPS 3	**1.92**	**1.20 to 3.10**	**0.007**
SOFA	1.01	0.66 to 1.55	0.952
Sex at birth (male)	0.82	0.55 to 1.21	0.316
Tuberculosis	0.87	0.51 to 1.48	0.601
Surgery			
Coagulation issue on surgery day^a^	**2.60**	**1.39 to 5.15**	**0.004**
Hemodialysis on surgery day	**2.47**	**1.61 to 3.82**	**< 0.001**
P/F ratio < 200 on surgery day	**2.62**	**1.62 to 4.35**	**< 0.001**
Postoperative complications	0.79	0.38 to 1.64	0.516

Bold: p-value < 0.05. CI: confidence interval; COVID-19: Coronavirus disease 2019 by SARS-CoV-2; OR: odds ratio; P/F: the ratio of arterial oxygen partial pressure to fractional inspired oxygen; SAPS 3: Simplified Acute Physiology Score 3. SOFA: sequential organ failure assessment score. TCT: tracheostomy. ^a^Coagulation issue was defined as: thrombocytopenia (< 100,000), or prolonged Prothrombin Time (PT) and/or activated Partial Thromboplastin Time (aPTT); or active bleeding and/or the need for plasma and/or platelet transfusions for the surgical procedure.

Given the high prevalence of COVID-19 in the study population, an additional multivariate logistic regression analysis was performed excluding patients with SARS-CoV-2 infection (Supplementary Table S1) to assess the robustness of the primary model. In this analysis, advanced age, diabetes mellitus, coagulopathy, and the need for hemodialysis on the day of tracheostomy remained independently associated with in-hospital mortality. Notably, when the SARS-CoV-2 subcohort was excluded, the presence of HIV infection emerged as a predictor of mortality, whereas the initiation or maintenance of combined antiretroviral therapy (cART) during ICU admission was associated with lower in-hospital mortality among these patients.

## Discussion

We analyzed the outcomes of patients who underwent tracheostomy for infectious diseases over a five-year period in a specialized infectious disease hospital. In this cohort, hospital mortality was high (64.6%) and was independently associated with advanced age, higher clinical severity at admission (SAPS 3 and SOFA scores), diabetes mellitus, coagulopathy, need for hemodialysis, and impaired oxygenation at the time of tracheostomy. These findings suggest that outcomes are primarily driven by systemic severity and organ dysfunction rather than by infectious etiology or procedural characteristics at the time of tracheostomy.

Tracheostomy has been traditionally associated with several potential benefits in patients requiring prolonged mechanical ventilation, including reduced need for sedation, faster weaning from ventilatory support, and shorter stays in both the ICU and the hospital^13,15^. The main challenges in managing these patients remain the appropriate identification of those who are most likely to benefit from the procedure within critically ill populations^14^.

The SAPS and SOFA scores are well-validated tools for assessing disease severity in intensive care and, in this study, their association with death reinforces their applicability among patients with an indication for tracheostomy^16,17^. A previous multicenter prospective study conducted in Brazil identified the SAPS score as an independent predictor of hospital mortality^18^. Our findings further emphasize the crucial role of overall clinical severity at the time of tracheostomy in determining patient outcomes. Higher values of these scores reflect greater organ dysfunction and higher risk of complications, directly impacting prognosis^19^.

Interestingly, other potentially relevant clinical conditions—such as chronic pulmonary disease, heart failure, neoplasms, and prior hemodialysis—were not significantly associated with mortality in this cohort. Although these variables are traditionally linked to increased clinical complexity, it is possible that the impact of acute infectious disease overshadowed the relative contribution of these comorbidities to the final outcome. This finding reinforces the notion that, in critically ill patients with severe infection, the acute pathophysiologic response and degree of organ dysfunction play a more decisive role in mortality than underlying chronic conditions^20^.

The analysis of infectious diseases associated with tracheostomy revealed that tuberculosis was the only infection significantly associated with survival in unadjusted comparisons, being more prevalent among survivors. This finding should be interpreted with caution, as all patients included in the study presented with acute or decompensated clinical conditions requiring ICU admission, and the observed association may reflect differences in baseline severity rather than a protective effect of tuberculosis itself. Although seemingly counterintuitive, this finding may reflect that some patients with tuberculosis already had an established diagnosis or were under treatment at the time of admission, possibly presenting with less acute organ dysfunction at the time of tracheostomy. Importantly, this association was not maintained in the multivariate model, indicating that tuberculosis was not an independent predictor of survival, but rather a marker of a different clinical presentation within the ICU.

A trend toward higher mortality was also observed among patients diagnosed with COVID-19, consistent with consolidated evidence from the pandemic period^21,22^. The COVID-19 literature reports high mortality and variable outcomes, although tracheostomy is considered safe when appropriately indicated^12,23^. SARS-CoV-2 infection was frequently associated with severe respiratory failure and prolonged mechanical ventilation, representing one of the main indications for tracheostomy during the study period. However, when COVID-19 was compared with other infectious etiologies, no statistically significant difference in hospital mortality was observed. The high prevalence of COVID-19 among both survivors and nonsurvivors may have reduced the discriminative power of this variable, preventing the difference from reaching statistical significance.

Given that COVID-19 accounted for a substantial proportion of the cohort, a sensitivity analysis excluding these patients was performed to explore the robustness of the findings. This approach was considered particularly relevant in the context of patients with underlying immunodeficiency, especially those with HIV infection (analyzed here as a baseline comorbidity), which represents a significant subgroup in this population with distinct immunological profiles. In this non–COVID-19 subgroup, the presence of HIV was identified as an independent marker of increased mortality, whereas the initiation or maintenance of combined antiretroviral therapy (cART) during ICU admission was associated with improved survival. This observation is consistent with previous evidence suggesting a survival benefit of initiating or maintaining antiretroviral therapy in critically ill people living with HIV^24^. These findings should be interpreted as complementary and exploratory, reflecting the potential influence of cohort composition on the observed associations, rather than definitive causal relationships. Taken together, these results suggest that, beyond infectious etiology alone, baseline immunological status and access to disease-specific therapy may play a relevant role in determining outcomes, alongside systemic severity and the adequacy of clinical management.

It is important to note that many patients in this study presented with more than one concurrent infectious disease. Coinfection is particularly common among immunosuppressed populations, such as people living with HIV, and also in critically ill patients, in whom fungal, bacterial, and viral infections may coexist^25,26^. This diagnostic overlap represents a limitation for the isolated analysis of each etiologic agent, as it may dilute the statistical association of some infections with clinical outcomes. Therefore, the results should be interpreted in light of the patients’ clinical and microbiological complexity.

Furthermore, the lack of statistically significant associations for several opportunistic or endemic infections—such as pneumocystosis^27^, cryptococcosis^28^, histoplasmosis^26^, and toxoplasmosis^29^—does not exclude their clinical relevance. In many cases, the low absolute frequency or concomitant presence of multiple infections may have limited the detection of significant differences.

Taken together, these results reinforce that, in the setting of tracheostomy for infectious diseases, hospital outcomes are more strongly related to systemic severity and selected comorbidities than to the infectious etiology itself^12,30^.

Our results highlight the determinant role of clinical severity at the time of tracheostomy as a factor associated with hospital mortality. Three clinical variables evaluated on the day of the procedure showed significant associations with mortality: coagulopathy, need for hemodialysis, and lower arterial oxygenation (PaO₂/FiO₂ ratio). All three were confirmed as independent predictors of mortality in the multivariate analysis.

Coagulopathy was significantly more frequent among patients who died. This finding aligns with previous literature describing hemostatic disorders as markers of systemic organ dysfunction in critically ill patients, in addition to directly increasing the risk of intraoperative and postoperative hemorrhagic complications^31,32^.

The need for hemodialysis on the day of tracheostomy was also strongly associated with mortality. Acute kidney injury is widely recognized as a marker of severity in ICU patients, often reflecting advanced multiorgan failure^33,34^. Dialysis dependence at the time of the procedure likely identifies a subgroup of patients with extreme physiological derangement and limited capacity for recovery. In this context, the shorter duration of mechanical ventilation, tracheostomy, and hospital stay observed among nonsurvivors should not be interpreted as a favorable outcome, but rather as a consequence of early death following the procedure. Consequently, such observations reflect differences primarily related to survival and should be viewed as descriptive outcomes of the nonsurvivor subgroup rather than valid comparative associations. These findings raise important considerations regarding the proportionality and expected benefit of tracheostomy in patients with profound organ dysfunction, in whom the procedure may offer limited impact on clinical trajectory.

Another relevant finding was the association between lower PaO₂/FiO₂ ratios—a marker of pulmonary oxygenation—and unfavorable outcomes. Persistent hypoxemia despite prolonged ventilatory support suggests the presence of severe lung injury, such as acute respiratory distress syndrome (ARDS), which is frequently associated with high mortality^3,35^.

In our cohort, the majority of tracheostomies were indicated for prolonged mechanical ventilation, reflecting the clinical severity of patients at the time of the procedure. Most tracheostomies were performed after approximately two weeks of endotracheal intubation, with a median time of 16 days, which is consistent with previously reported practice patterns^36,37^. In line with most published series, prolonged intubation represented the primary indication for tracheostomy, whereas emergency procedures were uncommon. Only four tracheostomies were performed as emergency interventions in our cohort, a finding comparable to previous reports^22^.

Considering that postoperative complications were infrequent in this cohort and occurred at similar rates among survivors and nonsurvivors, the high mortality observed is more likely attributable to the severity of the underlying disease and organ dysfunction rather than to the tracheostomy procedure itself. This elevated mortality rate, compared to other series^22,38^, likely reflects the profile of a tertiary referral center for infectious diseases, which manages a high burden of critically ill patients with advanced organ dysfunction and severe respiratory failure. Notably, we did not identify previous studies encompassing such an extended data collection period specifically focused on infectious diseases. Finally, while the absence of cause-specific mortality data for each individual case—a common limitation in large retrospective cohorts—precludes definitive conclusions regarding the direct contribution of the procedure, these findings reinforce that systemic clinical status remains the primary driver of patient outcomes.

Several limitations should be acknowledged. This analysis presents valuable real-world evidence derived from a large cohort treated at a tertiary infectious disease referral center in Brazil, providing an overview of clinical practice in a highly specialized context. Nonetheless, certain limitations should be considered, including its retrospective nature, single-institution scope, and dependence on information extracted from medical records. Additionally, the absence of a control group of patients who did not undergo the procedure precludes definitive conclusions regarding the overall impact of tracheostomy on survival. Furthermore, the lack of cause-specific mortality data limited the ability to distinguish between different causes of death, including respiratory and non-respiratory etiologies. Prospective studies and clinical trials are warranted to confirm these results and to establish evidence-based recommendations for the optimal management of tracheostomy in patients with severe infectious diseases. Subsequent research should also aim to better clarify the association between disease severity and outcomes, as well as the influence of other clinical variables on the prognostic stratification of these patients.

These findings have important implications for clinical practice and future research. Further studies are warranted to explore the potential benefits of prognostic stratification in this population, as well as its applicability to other diseases. In addition, clinical trials assessing optimized management strategies for respiratory failure due to infectious diseases requiring tracheostomy are needed to establish evidence-based guidelines to improve patient care.

Overall, this study expands the understanding of factors associated with mortality in patients undergoing tracheostomy in the context of infectious diseases. Mortality appeared to be more strongly associated with clinical severity and organ dysfunction than with infectious etiology. In a sensitivity analysis excluding patients with COVID-19, HIV infection and the use of combined antiretroviral therapy (cART) emerged as additional determinants of outcome, underscoring the prognostic relevance of immunological status and disease-specific treatment. These findings highlight the importance of an individualized, multiparametric prognostic assessment among patients undergoing tracheostomy, incorporating age, comorbidities, severity scores, and physiological parameters. Such an approach may provide a valuable framework to better characterize the clinical trajectory and the high mortality observed in this population of patients with infectious diseases.

## Methods

This is a retrospective, observational cohort study including all patients with laboratory-confirmed infectious diseases who underwent tracheostomy performed by the same surgeon, using a standardized surgical technique, in the Intensive Care Unit (ICU) of the Evandro Chagas National Institute of Infectious Diseases (INI), a referral center for infectious diseases located in Rio de Janeiro, Brazil. The study period spanned from May 2020 to April 2025. The study design and reporting followed the Strengthening the Reporting of Observational Studies in Epidemiology (STROBE) recommendations for observational research. Ethical approval was granted by the Research Ethics Committee of the Evandro Chagas National Institute of Infectious Diseases (INI/Fiocruz), Oswaldo Cruz Foundation, Brazil (CAAE: 66495523.0.0000.5262). All procedures involving human participants were performed in accordance with relevant institutional guidelines and regulations. The requirement for informed consent was waived by this Research Ethics Committee due to the retrospective nature of the study and the use of anonymized data. Medical record review was conducted between January 2, 2024, and June 2, 2025. The investigators were granted access to the complete medical records database exclusively for research purposes. Throughout data collection, any personal identifiers deemed unnecessary for the study were excluded to preserve patient anonymity. All data were handled in accordance with privacy regulations and ethical standards, ensuring that no identifiable information was used or disclosed at any stage of the research process.

### Patient population

The analysis included hospitalized adult patients (aged 18 years or older) with an active or previously documented infectious diseases diagnosed during the same hospitalization in which tracheostomy was performed. The diagnosis of infectious disease was based on clinical evaluation, supported by laboratory, microbiological, and/or imaging findings, according to standardized diagnostic criteria for each suspected condition.

The underlying infectious disease could have been identified prior to ICU admission, at the time of admission, or during the hospital stay, provided that it was documented in the medical records during the same admission in which tracheostomy was performed.

Patients had respiratory failure requiring mechanical ventilation, as well as neurological or cardiac dysfunction, or sepsis, and underwent tracheostomy for respiratory management. The infectious disease was considered part of the clinical context leading to critical illness and ICU admission, although it was not required to be the sole direct indication for mechanical ventilation or for tracheostomy. All infectious diseases included in the analysis were associated with acute or decompensated clinical conditions requiring ICU admission.

Exclusion criteria included tracheostomy performed for indications unrelated to infectious diseases, or incomplete medical records.

### Surgery

All tracheostomy procedures were performed using the open surgical technique, following a standardized approach. A single surgeon conducted all interventions at the patient’s bedside, within individual negative-pressure ICU rooms, under continuous sedation.

### Data collection

Data were retrospectively collected from patients’ medical records using a standardized data extraction form. The following baseline data were collected:

- **Demographic characteristics**: Body mass index (BMI), sex at birth, and age.
- **Comorbidities**: diabetes mellitus, cardiovascular disease, chronic pulmonary disease, prior hemodialysis, hypertension, malignant neoplasms and HIV infection.
- **Infectious diseases**: all infectious diseases identified in the study population.
- **Clinical status prior to tracheostomy**: Simplified Acute Physiology Score 3 (SAPS 3) and Sequential Organ Failure Assessment (SOFA) score at ICU admission; primary reason for ICU admission; and duration of mechanical ventilation prior to tracheostomy (collected for descriptive purposes only).
- **Clinical parameters on the day of tracheostomy**: Arterial oxygenation measured by the PaO₂/FiO₂ ratio (in mmHg), use of vasopressors, presence of coagulation disorders, and whether the patient was undergoing hemodialysis.

Coagulation disorders: Thrombocytopenia (platelet count < 100,000/mm³), prolonged prothrombin time (PT) and/or activated partial thromboplastin time (aPTT), active bleeding, and/or the need for transfusion of plasma or platelets for the procedure.

- **Intraoperative findings**: Complications and technical difficulties.
- **Postoperative outcomes**: Primary outcome: In-hospital mortality. Secondary outcomes: ICU mortality, procedure-related complications, total duration of mechanical ventilation (in days), number of days from tracheostomy to decannulation, decannulation status (yes/no), length of ICU stay, and overall length of hospital stay.

### Statistical analysis

Statistical analyses were performed using the R^®^ software for Microsoft Windows^®^. A *p*-value below 0.05, within a 95% confidence interval (CI), was considered indicative of statistical significance. Based on an estimated in-hospital mortality rate of 70%, a minimum sample size of 351 patients was calculated to achieve a 95% CI with a 5% margin of error.

Descriptive analysis was used to outline demographic and clinical characteristics. Continuous variables were summarized as medians and interquartile ranges (IQR), while categorical variables were expressed as absolute numbers (N) and percentages (%). In cases where the median of a continuous variable was zero, both the mean and standard deviation were additionally reported. Group comparisons for continuous variables were conducted using the Wilcoxon or Kruskal-Wallis tests, and categorical data were compared using Pearson’s chi-squared (χ²) test.

To identify independent predictors of in-hospital mortality, variables significantly associated with the primary outcome in univariate analyses (*p* < 0.05) were included in a multivariate logistic regression model. In addition, clinically relevant variables were retained based on their potential biological or epidemiological importance. To address multicollinearity, only predictors with a Variance Inflation Factor (VIF) < 5 were retained in the final analysis^39^. Continuous variables were categorized according to cut-off points defined by the Youden index, which identifies the threshold that maximizes the sum of sensitivity and specificity, to reduce the impact of outliers on outcome estimation. The performance of the final multivariate model was assessed using the Hosmer-Lemeshow goodness-of-fit test for calibration and the Area Under the ROC Curve (AUC) for discriminative power, providing complementary information on model performance. Regarding secondary outcomes such as ICU length of stay and decannulation, comparisons between groups were performed descriptively to avoid causal interpretations, acknowledging the inherent survival-related constraints of these variables.

Given that COVID-19 accounted for a substantial proportion of the study population, potentially influencing the associations observed in the primary analysis, an additional sensitivity analysis using multivariate logistic regression was performed excluding patients with SARS-CoV-2 infection (Supplementary Table S1) to assess the robustness of the findings and to explore whether the identified predictors remained consistent in a non-COVID cohort.

## Electronic Supplementary Material

Below is the link to the electronic supplementary material.

Supplementary Material 1

## Data Availability

All data from the current study are reported in the manuscript and tables. In addition, data are available upon reasonable request to André Accetta, the corresponding author, from the Evandro Chagas National Institute of Infectious Diseases, Oswaldo Cruz Foundation, Rio de Janeiro (RJ), Brazil.
