# Supplementary material for: Outcomes and mortality predictors after tracheostomy in intensive care unit patients with infectious diseases: A 5-year retrospective cohort study from Brazil
Source: Sci Rep. 2026 May 8;16:21101. doi: 10.1038/s41598-026-51458-2 (PMC13342089; doi:10.1038/s41598-026-51458-2)
Supplement: Supplementary file 1 — Supplementary Material 1 [file 41598_2026_51458_MOESM1_ESM.doc]

**Outcomes and Mortality Predictors After Tracheostomy in Intensive Care Unit Patients With Infectious Diseases: A 5-Year Retrospective Cohort Study From Brazil.**

**Supplementary Table S1: Multivariate logistic regression analysis of independent predictors associated with in-hospital mortality in a cohort of 243 ICU patients undergoing tracheostomy for infectious diseases in a referral center in Brazil (2020–2025), excluding patients with COVID-19.**

| FACTOR | OR | 95% CI | p-value |
| --- | --- | --- | --- |
| **Age >60 years** | **2.94** | **1.20 to 7.14** | **0.002** |
| **Diabetes** | **5.39** | **1.39 to 27.83** | **0.024** |
| Hypertension | 1.09 | 0.42 to 2.84 | 0.852 |
| **HIV infection** | **7.46** | **2.88 to 20.99** | **<0.001** |
| SAPS 3 | 1.00 | 0.97 to 1.04 | 0.834 |
| SOFA | 1.10 | 0.96 to 1.28 | 0.187 |
| Sex at birth (male) | 0.85 | 0.42 to 1.71 | 0.647 |
| Tuberculosis | 0.93 | 0.48 to 1.82 | 0.825 |
| **cART use** | **0.11** | **0.04 to 0.24** | **<0.001** |
| Surgery | | | |
| **Coagulation issue on surgery day^a^** | **2.58** | **1.05 to 6.77** | **0.044** |
| **Hemodialysis on surgery day** | **2.36** | **1.13 to 5.04** | **0.024** |
| P/F ratio < 200 on surgery day | 1.25 | 0.46 to 3.57 | 0.662 |
| Postoperative complications | 1.52 | 0.42 to 5.88 | 0.525 |
| **Bold: p-value <0.05.** cART: combined antiretroviral therapy initiated or maintained during hospitalization; CI: confidence interval; OR: odds ratio; P/F: the ratio of arterial oxygen partial pressure to fractional inspired oxygen; SAPS 3: Simplified Acute Physiology Score 3. SOFA: sequential organ failure assessment score. TCT: tracheostomy. ^a^ Coagulation issue was defined as: thrombocytopenia (<100,000), or prolonged Prothrombin Time (PT) and/or activated Partial Thromboplastin Time (aPTT); or active bleeding and/or the need for plasma and/or platelet transfusions for the surgical procedure. | | | |
